# Kinetic Estimated Glomerular Filtration Rate in Predicting Paediatric Acute Kidney Disease

**DOI:** 10.3390/jcm12196314

**Published:** 2023-09-30

**Authors:** Flavia Chisavu, Mihai Gafencu, Lazar Chisavu, Ramona Stroescu, Adalbert Schiller

**Affiliations:** 1 University of Medicine and Pharmacy ‘Victor Babes’, 300041 Timisoara, Romania; farkasflavia8@gmail.com (F.C.); chisavulazar@gmail.com (L.C.); ramona.giurescu@gmail.com (R.S.); schiller.adalbert@gmail.com (A.S.); 2 Louis Turcanu’ Emergency County Hospital for Children, 300011 Timisoara, Romania; 3 Centre for Molecular Research in Nephrology and Vascular Disease, Faculty of Medicine ‘Victor Babes’, 300041 Timisoara, Romania

**Keywords:** AKI, AKD, kinetic GFR, children, MAKE30

## Abstract

Kinetic estimation of glomerular filtration rate (KeGFR) has proved its utility in predicting acute kidney injury (AKI) in both adults and children. Our objective is to assess the clinical utility of KeGFR in predicting AKI severity and progression to acute kidney disease (AKD) in patients already diagnosed with AKI and to examine major adverse kidney events at 30 days (MAKE30). We retrospectively calculated the KeGFR within the first 24 h of identified AKI (KeGFR_1_) and in the 24 h prior to AKD (KeGFR_2_) in all admitted children under 18 years old. The cohort consisted of 803 patients with AKI. We proposed a new classification of KeGFR stages, from 1 to 5, and assessed the predictive value of KeGFR stages for AKD development and MAKE30. AKI severity was associated with lower KeGFRs. KeGFR_1_ and KeGFR_2_ predicted AKD with AUC values between 0.777 and 0.841 respectively, *p* < 0.001. KeGFR_2_ had the best performance in predicting MAKE30 (AUC of 0.819) with a sensitivity of 66.67% and specificity 87.7%. KeGFR_1_ stage 3, 4 and 5 increased the risk of AKD by 3.07, 6.56 and 28.07 times, respectively, while KeGFR_2_ stage 2, 3, 4 and 5 increased the risk of AKD 2.79, 3.58, 32.75 and 80.14 times. Stage 5 KeGFR_1_ and KeGFR_2_ stages 3, 4 and 5 increased the risk of MAKE30 by 7.77, 4.23. 5.89 and 69.42 times in the adjusted models. KeGFR proved to be a useful tool in AKI settings. KeGFR dynamics can predict AKI severity, duration and outcomes.

## 1. Introduction

Acute kidney injury (AKI) is a global health problem with a reported incidence of 26% in hospitalized children [1]. AKI is the reflection of the underlying disease severity. Some steps towards improving the outcomes for AKI are early identification of the individual risk factors, proper management and rapid intervention. The operational framework period for AKI that persists longer than 7 days provides time for intervention but at the cost of time-dependent nephron loss. Acute kidney disease (AKD) is defined as the link between AKI and chronic kidney disease (CKD), lasting between 7 to 90 days; thus AKD is a sequel of acute kidney injury (AKI) [2]. Current pediatric studies report AKD as an independent risk factor for progression to CKD [3,4,5]. The importance of AKD in clinical settings is poorly explored in the pediatric field [3,4,6,7,8]. AKD development and recovery are currently assessed using filtration markers, such as serum creatinine [2].

Kinetic estimated glomerular filtration rate (KeGFR) has been used in acute settings as part of a multidimensional approach for AKI prediction [9,10,11,12]. Kinetic modeling of eGFR can rapidly identify changes in kidney function any time during the first 24 h, proving its utility in intensive care unit settings [9,10,11,12]. In cardiac surgery and cardiac transplantation patients respectively, KeGFR proved to be correlated with AKI severity and mortality [9,10]. In one study in critically ill patients, the authors reported the AKI classification system and KeGFR to be complementary to each other, with a significant impact on RRT necessity and long-term survival [11]. Current studies have found KeGFR highly sensitive in predicting mostly severe AKI [10,12]. Although 10 years have passed since Chen introduced the KeGFR for cases where the plasma creatinine changes rapidly [13], only a few studies have addressed the utility of this tool in the clinical pediatric settings, and all of them were performed in intensive care units (ICUs) [9,12,14]. Also, there are no studies reporting on the importance of KeGFR in predicting AKD.

KeGFR is an adequate tool in assessing the risk of AKI in children, as the formula derives from the initial serum creatinine level, the distribution volume, the creatinine production rate and the given time differences of the two serum creatinine values [9]. Based on current data, we performed a retrospective study to assess the utility of KeGFR in predicting AKD and MAKE30 in pediatric AKI settings using personalized KeGFR for age- and height-corrected distribution volume of creatinine.

## 2. Material and Methods

A retrospective observational study was conducted in a tertiary care teaching and research hospital in western Romania from 1 of July 2014 to 31 December 2022. All patients under 18 years of age were screened based on changes in serum creatinine (SCr) via The Laboratory Information System and Hospital Information System. SCr measurement was performed using the Jaffe Abbott method. AKI was defined and staged using the 2012 Kidney Disease Improving Global Outcomes (KDIGO) SCr criteria [15]. AKD was defined and staged according to the 2017 ADQI consensus statement [2] on the 8th day of persistent AKI based on the SCr level. AKD is the persistence of AKI beyond day 7, regardless of AKI stage and AKI cause. AKD was classified based on the SCr value on the 8th day as follows: stage 1 AKD as a persistence of 1.5–1.9 times higher than baseline SCr; stage 2 as a persistence of 2–2.9 times higher; and stage 3 as persistence more than three times higher than baseline SCr or the necessity of renal replacement therapy (RRT). Chronic kidney disease (CKD) was defined according to the KDIGO CKD guidelines from 2012 [16]. AKI and AKD had been diagnosed based on an increase in SCr at a given time during hospitalization. Severe AKI was considered KDIGO stages 2 and 3. Severe AKD were all patients with stage 2 or 3 AKD. Exclusion criteria were a single measurement of SCr, end-stage kidney disease, patients who did not have their height and weight recorded, or a lack of two consecutive SCr measurements. Demographics, clinical characteristics, and outcomes were retrieved from data at the time of admission (day 1). Urine output measurement was not available for the entire cohort; thus we could not include urine output in our analysis.

Estimated glomerular filtration rate (eGFR) was calculated using the Schwartz formula [17] as follows:

$$\mathrm{eGFR}=\frac{k\;\times \;\left(\mathrm{height}\;\mathrm{in}\;\mathrm{cm}\right)}{\mathrm{SCr}\;\left(\frac{\mathrm{mg}}{\mathrm{dL}}\right)},$$

where *k* = 0.33 in preterm infants, *k* = 0.45 in infants at term to 1 year old, *k* = 0.55 in children over 1 year and under 13 years old, *k* = 0.55 for adolescent females 13–18 years old, and *k* = 0.7 for adolescent males 13–18 years old. KeGFR was calculated using Chen’s formula [13],

$$\mathrm{KeGFR}=\frac{baseline\;\mathrm{SCr}\times \mathrm{eGFR}}{Mean\;\mathrm{SCr}}\times 1-(\frac{24\times \Delta \mathrm{SCr}}{\Delta t\left(h\right)\times \mathrm{Max}\Delta \frac{\mathrm{SCr}}{\mathrm{day}}})$$

$$\mathrm{Max}\;\Delta \mathrm{SCr}/\mathrm{day}=\frac{baseline\;\mathrm{SCr}\times \mathrm{eGFR}\times 1.44}{VD(L)}$$

Baseline SCr was defined as the lowest SCr in the 3 months prior to AKI diagnosis, or the minimum inpatient SCr value during follow-up. Mean SCr was calculated from two consecutive SCr measurements. ΔSCr was the difference between the two SCr values, and Δt (h) was the interval in hours between two SCr measurements (maximum of 24 h). The maximum ΔSCr/day was defined as the change (increase) in SCr that occurred in a state of no kidney function dependent on volume of distribution (VD). The VD of creatinine is close to total body water, and it was estimated using Morgenstern et al.’s recalculation of Mellits and Cheek’s equation [18] based on height and sex as follows (Table 1):

After adjusting all data for age and sex, KeGFR was calculated twice, using two separate SCr set of values. KeGFR_1_ was calculated using the SCr levels on days 1 and 2 of identified AKI, and KeGFR_2_ was calculated from two SCr values in days 6 and 7, before progression to AKD. The cut-off values of KeGFR were chosen based on KDIGO CKD classification [13] as stage 1 (KeGFR > 90 mL/min/1.73 sm), stage 2 (KeGFR between 60–90 mL/min/1.73 sm), stage 3 (KeGFR between 30–60 mL/min/1.73 sm), stage 4 (KeGFR between 15–30 mL/min/1.73 sm) or stage 5 (KeGFR < 15 mL/min/1.73 sm).

Outcomes of AKI were evaluated via MAKE30 (major adverse kidney events within 30 days). MAKE was defined as death, necessity of RRT or the presence of AKD at day 30 after AKI documentation. The study was approved by the hospital’s Medical Ethics Committee in accordance with the Ethics Code of the World Medical Association and informed consent was waived.

### Statistical Analysis

Data are presented as median and percentage for non-normally distributed continuous variables and categorical ones, respectively. All continuous variables were tested for normality using the Shapiro–Wilk test. For normally distributed variables, we used independent *t*-test or ANOVA. For non-normally distributed continuous variables, the median and interquartile ranges (IQR) were reported, and groups were compared using the Wilcoxon Signed Ranks test. Discrete variables were analyzed with the chi-square test. Odds ratio (OR) and 95% confidence interval (95% CI) were calculated. In order to assess the independent factors that predict the risk of AKD and MAKE30 in our cohort, we employed a backward multivariable logistic regression model. An Akaike information criterion (AIC) was used in order to determine the best model. Receiver-operating characteristic curves (ROCs) were plotted to determine the prognostic values of KeGFR, and area under the curve (AUC) values were generated to determine the predictive values of KeGFR_1_ and KeGFR_2_. The following values were used to describe AUC-ROCs: 0.6–0.69, poor; 0.7–0.79, fair; and 0.8–0.89, good. In this study, a *p*-value of 0.05 was considered the threshold for statistical significance. Data were analyzed using MedCalc Statistical software version 22.009. MedCalc^®^ Statistical Software version 22.009 (MedCalc Software Ltd, Ostend, Belgium; https://www.medcalc.org; accessed on 25 August 2023)

## 3. Results

The final cohort consisted of 803 patients with AKI that met the inclusion criteria. We divided the cases into two groups: the AKD group (219 patients) and the non-AKD group (585 patients). Baseline characteristics are presented in Table 2.

**Figure 1:**
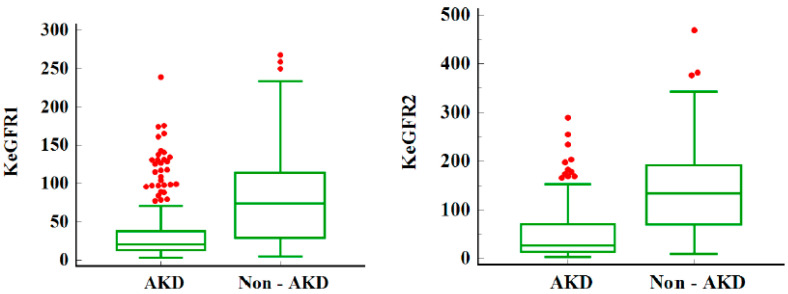
KeGFR in children with AKD and non-AKD. Legend: Median and IQR for KeGFR in children with AKD and non-AKD; KeGFR_1_ and KeGFR_2_ are lower in the AKD group; AKD = acute kidney disease; KeGFR = kinetic estimated glomerular filtration rate in mL/min/1.73 s. The red dots represent the outliers—data points that are located outside the whiskers of the box plot.

There were no differences between sexes. Patients that progressed to AKD were younger, namely, 3 days versus 365 days (*p* < 0.0001). The prevalence of severe AKI was higher in the AKD group (88.5%) as compared to the no AKD group. Patients that developed AKD maintained the same trend, with severe AKD in 72.8% of the patients (stage 2 or 3 AKD). Prerenal AKI was the main cause of AKI. Patients with intrinsic AKI were more prone to developing AKD as compared to other AKI etiologies. Intrinsic AKI could be traced in 28% of AKD patients and only in 15.6% of the non-AKD ones. As expected, baseline SCr was higher and eGFR lower in the AKD group (*p* < 0.0001). KeGFR_1_ and KeGFR_2_ were 3.57 times and 4.99 times lower, respectively, in the AKD group—see Figure 1. Out of 803 patients, only 16 required RRT (2%), with the highest rate of RRT in the AKD group (12 patients). Non-AKD patients expressed lower rates of pre-existent CKD. The overall mortality was 6.6% (53 patients) without significant statistical differences between AKD and non-AKD groups.

We identified a strong correlation between KeGFR_1_ staging and AKI severity and causes—see Table 3 (*p* < 0.0001). KeGFR was classified in KeGFR stages as described in Methods. Within the first 24 h of identified AKI, almost half the cases (45%) were stage 1 or 2 (KeGFR > 60 mL/min/1.73 sm). Regarding AKI stages, AKI stage 1 had the highest KeGFR (stages 1 and 2) as compared to severe AKI (stages 2 and 3). As severity of AKI increased, KeGFR stage increased. In patients that progressed to AKD (217 patients out of 803), severe AKD was also associated with higher KeGFR stages.

After evaluating KeGFR within the first 24 h of identified AKI, we performed the second analysis of KeGFR_2_ on day 7 using SCr levels from days 6 and 7 in Table 4. KeGFR_2_ was calculated for 310 patients out of whom 151 children developed AKD (48.7%). The KeGFR_2_ distribution was similar to KeGFR_1_, with half the patients having a KeGFR > 60 mL/min/1.73 sm (stages 1 and 2). This analysis once again confirms that AKI severity is associated with higher stages of KeGFR_2_. Also, AKD severity is associated with decreased levels of KeGFR_2_. In the subgroup of patients with KeGFR2, we evaluated the distribution of AKI causes. As previously mentioned, a prerenal cause of AKI was the leading one in all KeGFR groups. The results from KeGFR_1_ and KeGFR_2_ showed that more than 60% of patients with intrinsic AKI had a KeGFR > 60 mL/min/1.73 sm.

We performed a logistic regression model with AKD-dependent variables and KeGFR_1_ stages as independent ones (Supplemental Table S1). The unadjusted model proved to be a good one (Nagelkerke R^2^ = 0.27), with an AUC of 0.763. KeGFR_1_ stages 3, 4 and 5 increased the risk of AKD development by 2.76, 5.33 and 27.23 times, respectively. After adjusting the regression model for sex, AKI stage and AKI causes, the model improved (Nagelkerke R^2^ = 0.32, AUC = 0.801), with KeGFR_1_ increasing the incidence of AKD by 3.07, 6.56 and 28.07 times in stages 3, 4 and 5, respectively.

The logistic regression models with AKD-dependent variables and KeGFR_2_ stages as independent ones proved to be a good fit in both unadjusted and adjusted (Nagerkerke R^2^ 0.43 and 0.44, AUC = 0.809 and 0.827, respectively). In the unadjusted model, KeGFR_2_ stages 2, 3, 4 and 5 increased the risk of AKD by 2.65, 3.03, 28.72 and 70.09 times, respectively, and in the adjusted one by 2.79, 3.58, 32.75 and 80.14, respectively (Supplemental Table S2).

MAKE30 of the entire cohort revealed 111 events (13.8%): 53 deaths, 11 patients requiring RRT and 47 children with AKD. Logistic regression with MAKE30 as a dependent variable and KeGFR_1_ stages proved to be a good fit in both unadjusted and adjusted models (Nagerkerke R^2^ = 0.18 and 0.23, AUC = 0.675 and 0.764, respectively). Only stage 5 KeGFR_1_ increased the risk of MAKE30 by 10.42 times in the unadjusted model and by 7.77 times in the adjusted one (Supplemental Table S1). KeGFR_2_ stages 3, 4 and 5 increased the risk of MAKE30 by 2.8, 4.48 and 43.17, respectively, in unadjusted model and by 4.23, 5.89 and 69.42, respectively, in the adjusted one (Nagelkerke R^2^ 0.37 and 0.43, AUC = 0.809 and 0.837, respectively) (Supplemental Table S2).

**Figure 2:**
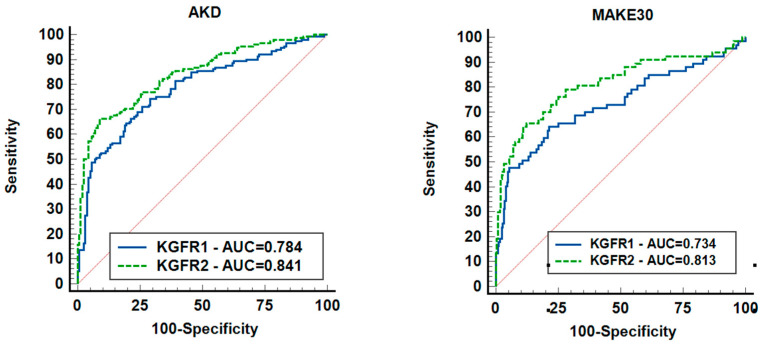
ROCs showing the ability of KeGFR to predict AKD and MAKE30. Legend: AKD = acute kidney disease; KeGFR = kinetic estimated glomerular filtration rate; MAKE30 = major adverse kidney events; 310 patients were included.

KeGFR calculation within the first 24 h of documented AKI, as well as 24 h prior to AKD, predicted AKD development and MAKE30—see Figure 2. KeGFR_1_ and KeGFR_2_ predicted AKD with AUC values between 0.777 (95% CI 0.747–0.806) and 0.841 (95% CI 0.795–0.88), *p* < 0.001. KeGFR_2_ had the best performance in predicting AKD, with 66.45% and specificity 91.14% with associated criterion ≤ 40.41 mL.min/1.73 sm, as well as in predicting MAKE30 with an AUC of 0.819 (95% CI 0.772–0.861) with a sensitivity of 66.67% and specificity 87.7% with associated criterion ≤ 25.69 mL/min/1.73 sm, *p* < 0.001. It should be mentioned that the ROC-AUC of KeGFR_2_ included 310 patients. KeGFR_1_ had a fair performance in predicting AKD AUC of 0.777 with a sensitivity of 76.15% and specificity of 68.55%, with associated criterion ≤ 39.18 mL/min/1.73 sm. KeGFR_1_ included all patients (N = 803). KeGFR_1_ had an AUC of 0.7 (95% CI 0.667–0.731) with an associated criterion of ≤21.28 mL/min/1.73 sm (*p* < 0.001), a 55.86% sensitivity and 81.65% specificity in predicting MAKE 30.

## 4. Discussion

In this study, KeGFR was analyzed for the first time as a predictor of AKD in children. To best of our knowledge, there are no studies that used KeGFR for AKD prediction in pediatric or adult settings. KeGFR has been used in predicting AKI in high-risk populations [9,10,11,12,14,19,20,21] with a high sensitivity and specificity. Until now, KeGFR has been used as a predictor of AKI; thus, it was calculated prior to AKI occurrence. The retrospective nature of our study allowed us to calculate KeGFR within the first 24 h of identified AKI as well as 24 h prior to AKD using the patient’s baseline SCr measurements. For better estimation of KeGFR, we employed Morgenstern et al.’s recalculation of Mellits and Cheek’s [18] formula to calculate the total body water. As the distribution volume of creatinine is close to total body water, we individualized this equation in order to eliminate any bias. Unlike in adults, any changes in total body water in children will influence SCr levels. Also, to reduce bias, we used Chen’s formula without the correction factor of 1.5 mg/dL corresponding to the maximum 24 h SCr increments in anuric patients with the estimation of maximum 24 h ΔSCr adjusted for individual distribution volume.

Our results showed that 1 in 4 children progressed to AKD (27.14%), hence the importance of predicting AKD. Progression to AKD is a cumulative result of medical intervention and underlying disease severity. As previously reported by KDIGO guidelines in 2012 and the ADQI consensus report, AKD is a sequel of AKI with increased morbidity and mortality with or without progression to CKD [2,15]. With a high progression rate from AKI to AKD, there is an imperative need for new and cheap tools to predict AKD occurrence. In our cohort, children from the AKD group were younger, with higher SCr levels from day 1 to day 7 of identified AKI reflected in lower KeGFR during the documented AKI episode. Our study showed that half the patients had a KeGFR < 60 mL/min/1.73 sm, reflecting AKI severity and AKI duration, consistent with previously reported pediatric data [9,12]. Besides reflecting AKI severity, reduced KeGFR levels were associated with AKD severity. Our results emphasize the importance of KeGFR_1_ calculation as a predictor of AKI persistence, as 70% of the children that developed AKD had a KeGFR below 60 mL/min/1.73 sm. AKI evolution is dependent on medical intervention (fluid resuscitation, nephrotoxic medication, fluid overload and need for mechanical ventilation) that in most cases leads to renal recovery. However, more than 25% of our patients had a prolonged AKI episode that further contributed to kidney injury. KeGFR_1_ proved there is a link between reduced day 1 and 2 renal function and AKD development as an independent variable. As expected, KeGFR_2_ had a higher prediction rate of AKD compared to KeGFR_1_. Given the medical intervention during the timeframe between the first 24 h of identified AKI and the 24 h prior to AKD development, KeGFR_2_ proved once again to be an independent risk factor for AKD. For example, a patient with KeGFR_1_ stage 5 (<15 mL/min/1.73 sm) has a 28-fold higher risk of progressing to AKD; hence, one could use KeGFR_2_ in dynamic for predicting the severity of AKD as well as for the risk of MAKE30. KeGFR_2_ is a better predictor for AKD and MAKE30 as a result of prolonged kidney injury. More than 70% of the patients with AKD had a KeGFR_2_ below 60 mL/min/1.73 sm, reflecting the transition from AKI state to AKD. The risk of AKD and MAKE30 starts from KeGFR_2_ stage 3 (<60 mL/min/1.73 sm) as opposed to stage 5 KeGFR_1_ (<15 mL/min/1.73 sm). With these results, we highlight the utility of repeated kinetic measurements. We suggest careful renal function monitoring in all AKI patients, especially in those with KeGFR_1_ stage 5 (<15 mL/min/1.73 sm) and KeGFR_2_ stages 3, 4 and 5 (<60 mL/min/1.73 sm), using repeated SCr measurements.

In clinical settings, several markers proved to be effective in predicting AKI, including KeGFR. For instance, Dewitte et al. [22] showed that KeGFR had a better performance in predicting AKI compared to plasma neutrophil gelatinase-associated lipocalin (NGAL), renal resistive index and product between tissue inhibitor of metalloproteinase-2 and urine insulin-like growth factor-binding protein 7. Hekmat et al. found KeGFR a better AKI predictor compared to other creatinine-based formulas with a strong correlation between KeGFR and NGAL [23]. While it is counterproductive to use specific and expensive biomarkers of kidney injury in clinical practice, in this study, we underline the utility of KeGFR as a cheap and handy tool.

Current studies have failed to find a significant relationship between reduced KeGFR and 30-day mortality [11,24], while Dewitte showed a good prediction of MAKE using KeGFR based model [22] in ICU settings. Our results showed there is a link between reduced KeGFR and a high risk of MAKE30 development. The KeGFR_2_ model proved to be superior compared to KeGFR_1_, proving the utility of KeGFR calculation once again in predicting worse renal outcomes. AKI evolution is marked by several outcomes: rapid renal recovery with return to baseline SCr, delayed recovery (up to 90 days), and progression towards CKD or death. When renal recovery does not occur during hospitalization, with progression to AKD, the second KeGFR calculation (days 6 and 7 after AKI diagnosis) proved to be a better predictor of worse renal outcome (AKD development, mortality and AKD at 30 days).

The KeGFR_1_ model proved to be fair in predicting MAKE30, with an AUC of 0.7, while the KeGFR_2_ model proved to be good, with an AUC of 0.819. For instance, Dewitte found a similar predictive value of KeGFR for MAKE in the adult population, with an AUC of 0.81 [22].

In our opinion, when SCr increases rapidly, kinetic modeling of GFR proves to be a better predictor of AKI outcomes, AKD development and worst renal outcomes at 30 days. One should mention the clinical usefulness of KeGFR, regardless the time of calculation, in both AKI-identified patients and in at-risk AKI patients, as previously reported (i.e., in ICU settings) [9,12,14].

The strength of this study is supported by the high number of AKI cases from a mixed pediatric population, both critically ill and non-critically ill, including neonates. Our electronic data system allowed us to identify baseline SCr, making it the first study to include baseline SCr and baseline eGFR in kinetic estimation formula in children, and to include all patients with consecutive SCr measurements within the first 24 h of identified AKI and the 24 h prior to AKD. In addition, we were able to adjust the formula of KeGFR to the maximum daily creatinine production using Morgenstern et al.’s recalculation of Mellits and Cheek’s equation based on height and sex. The strongest point was the use of this tool in order to predict AKD and MAKE30. The limitations were the lack of urine output criteria, single center and retrospective design.

Kinetic modeling of eGFR can be used as a complementary tool in AKI settings. KeGFR has already been validated as a severe AKI predictor providing a good prognostic and diagnostic approach. There is an imperative need for prospective studies in the pediatric population in order to validate our results. We suggest that KeGFR measurements could be employed in pediatric intensive care units for risk stratification in patients with or without AKI.

## Figures and Tables

**Table 1:** Total body water formula adjusted for age and sex.

Infants from 0 to 3 Months	0.887 × (Weight)^0.83^
Children 3 months to 13 years	0.0846 × 0.95 (if female) × (Height × Weight)^0.65^
Children over 13 years	0.0758 × 0.84 (if female) × (Height × Weight)^0.69^

Height = centimetres; Weight = kilograms.

**Table 2:** Baseline characteristics of the entire cohort.

Parameter		Total N = 803	AKD N = 218	Non-AKD N = 585	*p*-Value
Age (days)		330 (1–2920)	3 (1–3285)	365 (1–2555)	<0.0001
Sex (female)		387 (48.2%)	107 (49.1%)	280 (47.9%)	0.714
AKI stage	Stage 1	205 (25.5%)	25 (11.5%)	180 (30.8%)	<0.0001
	Stage 2	248 (30.9%)	52 (23.8%)	196 (33.5%)	
	Stage 3	350 (43.6%)	141 (64.7%)	209 (35.7%)	
AKD stage	Stage 1	-	59 (27.2%)	-	
	Stage 2	-	58 (26.7%)	-	
	Stage 3	-	100 (46.1%)	-	
AKI cause	Prerenal	630 (78.5%)	152 (69.7%)	478 (81.7%)	0.0002
	Renal	152 (18.9%)	61 (28%)	91 (15.6%)	
	Postrenal	21 (2.6%)	5 (2.3%)	16 (2.7%)	
LOS		12 (6–27)	25 (14.25–44.75)	9 (5–19)	<0.0001
Baseline SCr (mg/dL)		0.31 (0.21–0.45)	0.38 (0.24–0.54)	0.29 (0.21–0.4)	<0.0001
Baseline eGFR (mL/min/1.73 sm)		127.3 (67.07–189.36)	70.35 (40.21–134.32)	143.36 (84.56–200.89)	<0.0001
SCr day1 (mg/dL)		0.85 (0.61–1.11)	1.06 (0.8–1.58)	0.76 (0.55–0.99)	<0.0001
SCrday2 (mg/dL)		0.69 (0.45–0.97)	1.04 (0.76–1.64)	0.57 (0.39-0.79)	<0.0001
KeGFR1 (mL/min/1.73sm)		49.36 (21.71–103.59)	20.77 (13.49–38.04)	74.27 (29.27–114.3)	<0.0001
SCr day 6 (mg/dL) ^a^		0.58 (0.38–1.15)	0.98 (0.61–1.83)	0.4 (0.28–0.56)	<0.0001
SCr day 7 (mg/dL) ^a^		0.53 (0.35-1.02)	0.9 (0.57–1.83)	0.36 (0.25–0.49)	<0.0001
KeGFR2 (mL/min/1.73 sm) ^a^		70.72 (26.19–153.81)	26.75 (13.66–70.32)	133.66 (70–191.85)	<0.0001
RRT		16 (2%)	12 (5.5%)	4 (0.7%)	<0.0001
Pre-existent CKD		58 (7.2%)	24 (11%)	34 (5.8%)	0.0121
Deaths		53 (6.6%)	18 (8.3%)	35 (6%)	0.2487

Data are represented as median and IQR for continuous variables and as percentage for categorical data; N = total number, AKI = acute kidney injure; AKD = acute kidney disease; CKD = chronic kidney disease; LOS = length of stay; SCr = serum creatinine; RRT = renal replacement therapy; eGFR = estimated glomerular filtration rate; KeGFR = kinetic estimated GFR; sm = square meters; mg/dL = milligrams/deciliter; ^a^ included 310 patients.

**Table 3:** KeGFR_1_ (derived from days 1 and 2 SCr values) staging and distribution in AKI and AKD stages and AKI causes.

	KeGFR_1_ Stage 1 N = 263	KeGFR_1_ Stage 2 N = 100	KeGFR_1_ Stage 3 N = 138	KeGFR_1_ Stage 4 N = 212	KeGFR_1_ Stage 5 N = 90	*p*-Value
AKI stage 1	128 (48.7%)	27 (27%)	21 (15.2%)	25 (11.8%)	4 (4.4%)	<0.0001
AKI stage 2	102 (38.8%)	37 (37%)	46 (33.3%)	58 (27.4%)	5 (5.6%)	
AKI stage 3	33 (12.5%)	36 (36%)	71 (51.4%)	129 (60.8%)	81 (90%)	
AKD stage 1 *	17 (68%)	3 (27.3%)	15 (45.5%)	19 (23.7%)	5 (7.4%)	<0.0001
AKD stage 2 *	6 (24%)	3 (27.3%)	7 (21.2%)	26 (32.5%)	16 (23.5%)	
AKD stage 3 *	2 (8%)	5 (45.4%)	11 (33.3%)	35 (43.7%)	47 (69.1%)	
Prerenal AKI	187 (71.1%)	73 (73%)	113 (81.9%)	191 (90.1%)	66 (73.3%)	<0.0001
Intrinsic AKI	71 (27%)	21 (21%)	20 (14.5%)	18 (8.5%)	22 (24.4%)	
Postrenal AKI	5 (1.9%)	6 (6%)	5 (3.6%)	3 (1.4%)	2 (2.2%)	

AKI = acute kidney injury; AKD = acute kidney disease; KeGFR = kinetic estimated glomerular filtration rate; * included 217 patients with AKD. The percentages refer to the percentages within each KeGFR stage of each kidney disease class: AKI stages, AKD stages and AKI cause.

**Table 4:** KeGFR_2_ (derived from days 6 and 7 SCr values) staging and distribution in AKI and AKD stages and AKI causes.

	KeGFR_2_ Stage 1	KeGFR Stage 2 N = 32	KeGFR Stage 3 N = 51	KeGFR Stage 4 N = 47	KeGFR Stage 5 N = 43	*p*-Value
	N = 137					
AKI stage 1	41 (29.9%)	4 (12.5%)	4 (7.8%)	5 (10.6%)	1 (2.3%)	<0.0001
AKI stage 2	52 (38%)	12 (37.5%)	14 (27.5%)	5 (10.6%)	3 (7%)	
AKI stage 3	44 (32.1%)	16 (50%)	33 (64.7%)	37 (78.8%)	39 (90.7%)	
AKD stage 1 *	15 (50%)	9 (64.3%)	7 (29.2%)	1 (2.4%)	0 (0%)	<0.0001
AKD stage 2 *	10 (33.3%)	1 (7.1%)	10 (41.6%)	14 (33.3%)	4 (9.8%)	
AKD stage 3 *	5 (16.7%)	4 (28.6%)	7 (29.2%)	27 (64.3%)	37 (90.2%)	
Prerenal AKI	76 (55.5%)	18 (56.2%)	42 (82.4%)	34 (72.3%)	29 (67.4%)	0.0213
Intrinsic AKI	53 (38.7%)	11 (34.4%)	9 (17.6%)	13 (27.7%)	12 (27.9%)	
Postrenal AKI	8 (5.8%)	3 (9.4%)	0 (0%)	0 (0%)	2 (4.7%)	

AKI = acute kidney injury; AKD = acute kidney disease; KeGFR = kinetic estimated glomerular filtration rate; The model included 310 patients; * 151 having AKD. The percentages refer to the percentages within each KeGFR stage of each kidney disease class: AKI stages, AKD stages and AKI cause.

## Data Availability

The data collected for this study will be available for others, at request directly to the corresponding author. The data that will be available is represented by deidentified participant data. The inform consent form and statistical analysis plan will be available at request. The data will be available with publication. The data will be available at request at the e–mail address chisavulazar@gmail.com. The data will be shared after direct request and after approval of the proposal by all the authors.

## Supplementary Materials

The following supporting information can be downloaded at: https://www.mdpi.com/article/10.3390/jcm12196314/s1, Table S1. Logistic regression with AKD and MAKE30 dependant variable with KeGFR_1_ stages; Table S2. Logistic regression with AKD and MAKE30 dependant variable with KeGFR_2_ stages.
