# Supplementary material for: Kinetic Estimated Glomerular Filtration Rate in Predicting Paediatric Acute Kidney Disease
Source: J Clin Med. 2023 Sep 30;12(19):6314. doi: 10.3390/jcm12196314 (PMC10573153; doi:10.3390/jcm12196314)
Supplement: Supplementary file 1 [file jcm-12-06314-s001.zip › jcm-2616246-supplementary.pdf]

Supplementary Table S1. Logistic regression with AKD and MAKE30 dependant variable with $KeGFR_1$ stages.

Parameter	AKD	AKD adjusted	MAKE30	MAKE30 adjusted
$KGFR_1$ stage 1	Baseline	Baseline	Baseline	Baseline
$KGFR_1$ stage 2	-	-	-	-
$KGFR_1$ stage 3	2.76 (1.64-4.64)	3.07 (1.74-5.39)	-	-
$KGFR_1$ stage 4	5.33 (3.44-8.28)	6.56 (3.92-10.98)	-	-
$KGFR_1$ stage 5	27.23 (15.11-49.07)	28.07 (14.23-55.34)	10.42 (6.41-16.93)	7.77 (4.53-13.32)
Nagelkerke R^2	0.27	0.32	0.18	0.23
AUC	0.763	0.801	0.675	0.764

Legend: Adjusted for: sex, AKI stage, AKI cause; AKD=acute kidney disease; MAKE30=major adverse kidney events in the first 30 days; KeGFR=kinetic estimated glomerular filtration rate; AUC=area under the curve.

Supplementary Table S2. Logistic regression with AKD and MAKE30 dependant variable with $KeGFR_2$ stages.

Parameter	AKD	AKD adjusted	MAKE30	MAKE30 adjusted
$KGFR_2$ stage 1	Baseline	Baseline	Baseline	Baseline
$KGFR_2$ stage 2	2.65 (1.18-5.94)	2.79 (1.23-6.33)	-	-
$KGFR_2$ stage 3	3.03 (1.53-6)	3.58 (1.76-7.29)	2.8 (1.1-7.09)	4.23 (1.56-15.02)
$KGFR_2$ stage 4	28.72 (10.46-78.85)	32.75 (11.7-91.68)	4.48 (1.86-10.81)	5.89 (2.31-15.02)
$KGFR_2$ stage 5	70.09 (16.04-306.29)	80.14 (18.08-355.11)	43.17 (17.21-108.26)	69.42 (24.84-193.97)
Nagelkerke R^2	0.43	0.44	0.37	0.43
AUC	0.809	0.827	0.809	0.837

Legend: Adjusted for: sex, AKI stage, AKI cause; AKD=acute kidney disease; MAKE30=major adverse kidney events in the first 30 days; KeGFR=kinetic estimated glomerular filtration rate; AUC=area under the curve.
